# Towards the Elimination of Syphilis in a Small Developing Country

**DOI:** 10.1155/2015/801437

**Published:** 2015-07-28

**Authors:** Kameel Mungrue, Jeffrey Edwards, Azizah Fyzul, Billy Boodhai, Adita Narinesingh, Shasta Nanlal

**Affiliations:** ^1^Department of Paraclinical Sciences, Faculty of Medical Sciences, EWMSC, Mount Hope, Trinidad, Trinidad and Tobago; ^2^Queen's Park Counselling Centre, Charlotte Street, Port of Spain, Trinidad, Trinidad and Tobago

## Abstract

*Objective*. To describe the current epidemiological features of syphilis and congenital syphilis in Trinidad, 2009–2012. *Methods*. All laboratory confirmed syphilis cases diagnosed through a vertical program in the Ministry of Health, between 1/1/2009 and 31/12/2012, were identified. All relevant data were collected including address which was geocoded and mapped using ArcGIS 10.0 (Esri). Both spatial techniques and standardized incidence ratios were used to determine hot spots. *Results*. The annual cumulative incidence rate for syphilis remains high varying from 39 per 100 000 population in 2009 to 29 per 100 000 in 2012. We identified 3 “hot spots,” in urban areas of Trinidad. Young men and particularly young women in childbearing age 15–35 living in urban high density populations were commonly infected groups. *Conclusion*. The incidence of syphilis continues to be very high in Trinidad. New initiatives will have to be formulated in order to attain the global initiative to eradicate syphilis by 2015.

## 1. Introduction

In 2007, the World Health Organization (WHO) launched its Initiative for the Global Elimination of Congenital Syphilis, with the goal that by 2015 at least 90% of pregnant women are tested for syphilis and at least 90% of seropositive pregnant women receive adequate treatment [1]. Subsequently in September 2010, PAHO member states approved a strategy and plan of action for the elimination of mother-to-child transmission (MTCT) of HIV and congenital syphilis by resolution 50/12 at the 50th directing council meeting [2, 3]. One of the three targets set was “reduce the incidence of congenital syphilis to 0.5 cases or less per 1,000 live births by 2015.” The Caribbean community (CARICOM) health ministers endorsed this initiative [4].

In adults syphilis is a complex sexually transmitted disease that has a highly variable clinical course. Syphilis is defined mainly by its clinical presentation with modifications for surveillance purposes [5]. Clinically the primary lesion (chancre) presents mainly as an anogenital ulcer that appears 9–90 days after exposure, although extra-anogenital sites such as the lip, tongue, and tonsils from oral sex and kissing, nipple from kissing or wet nursing of infected babies, and finger with minor abrasion from touching infectious lesions can occur. Secondary syphilis presents with generalised rash affecting the palms and soles, generalised lymphadenopathy, and orogenital mucosal lesions, including snail tract ulcers and condylomata lata. Tertiary syphilis includes gummatous, cardiovascular, and neurological involvement. Gummatous syphilis (sometimes known as benign tertiary syphilis) can involve organs or supporting structures and can result in infiltrative or destructive lesions leading to granulomatous lesions or ulcers (e.g., skin) or perforation/collapse of structure (e.g., palate and nasal septum) or organomegaly. Gumma of the tongue may be prone to leukoplakia leading to malignant change. Late neurosyphilis can cause meningovascular syphilis leading to stroke syndromes and parenchymal involvement leading to general paresis and tabes dorsalis. Cardiovascular syphilis involves the aortic arch, which can lead to angina from coronary ostitis, aortic incompetence, and aortic aneurysm.

There is sparse data reflecting the true occurrence of congenital syphilis (CS) in Trinidad. In 1990 Ali reported that during the period from January 1985 to December 1988, 28 cases of CS were diagnosed shortly after birth at one major hospital in Trinidad with an average annual incidence of 1.5 per 1000 live births, seen at that particular institution [6]. Subsequently PAHO reported 45 cases of congenital syphilis in 2009 for the entire country, a rate of 2.3 per 1000 live births; both pieces of evidence suggest that CS remains a public health challenge [7]. Diagnosis and prevention of mother-to-child transmission of syphilis (MTCT) are feasible, inexpensive, and cost-effective in nearly every situation evaluated [8]. In fact congenital syphilis is preventable if a single dose of 2.4 million units of benzathine penicillin G is given to a pregnant woman with the infection during the first two trimesters of pregnancy [9–11]. The challenge is to find such women.

In order to monitor progress towards the goals of the elimination initiative in the region, PAHO requested that member countries gather information on several key programmatic elements. In Trinidad there are two tier systems of health care both operating independently of each other, a Public Health Care System (PHCS), and a Private Health Care System (PrHCS). The PHCS is funded by the Ministry of Health (MoH) and therefore all services are free as opposed to the PrHCS which is based on a fee for service model. The largest provider for the diagnosis and treatment of sexually transmitted infections is the MoH through a dedicated vertical program delivered at the Queen's Park Counselling Centre (QPCC). The laboratory services delivered by this program extend to PrHCS and therefore offer a reliable source of data from which syphilis occurrence in Trinidad and can be estimated. It is also a central repository for confirmatory testing.

The aim of this study therefore is to describe the current status of acquired syphilis in Trinidad, trends over the first decade of the 21st century, and a spatial analysis of syphilis cases, to inform strategies for achieving the elimination of syphilis.

## 2. Methods

We used a retrospective study design. The QPCC is a repository for all clinical and laboratory data for syphilis for the entire population of 1.3 million. Thus all clinical cases and laboratory investigations associated with a diagnosis of syphilis or CS were eligible for entry into the study. We defined syphilis and CS using the CDC surveillance case definitions, which include clinical features as well as a laboratory diagnosis. In particular for syphilis clinical features included one or more of the following: chancres consistent with primary syphilis, localized or diffuse mucocutaneous lesions often associated with generalized lymphadenopathy and, a reactive serologic test (nontreponemal: Venereal Disease Research Laboratory (VDRL) or treponemal (fluorescent treponemal antibody absorbed)). Similarly for CS the clinical features occur in an infant or child such as hepatosplenomegaly, rash, condyloma lata, snuffles, jaundice (nonviral hepatitis), pseudoparalysis, anaemia, or oedema (nephrotic syndrome and/or malnutrition) and stigmata (e.g., interstitial keratitis, nerve deafness, anterior bowing of shins, frontal bossing, mulberry molars, Hutchinson teeth, saddle nose, rhagades, or Clutton joints) in older children [12]. This was followed by laboratory confirmation using the* Treponema pallidum* particle agglutination assay (TPPA).

The results of all serum samples tested by the laboratory for VDRL between 2009 and 2012 were reviewed. VDRL positive samples using standard methods and quantitative VDRL testing were eligible for entry into the study. A positive VDRL supported by a positive confirmatory test was used to tract the clinical records to ensure that a case of syphilis met the case definitions. From the clinical records, data on age, gender, ethnicity, and address (without street number) were collected. In order to preserve strict confidentiality no data were collected that could link the data being collected to the client. We also used secondary data published by the MoH for the period 1994–2009 to demonstrate trends. This data reports only the recorded number of cases for each calendar year and published by the MoH in the annual reports of the MoH [13].

The street addresses of established syphilis cases diagnosed, between 01/01/2010 and 31/12/2012 only, were collected and mapped using ArcMap 10.0 GIS software (ESRI, Redlands, CA, USA). The geocoded addresses were transformed into a density map, using the Spatial Analyst Kernel Density Tool areas with the highest density of syphilis (defined as areas with greater than ten cases per square mile over the three-year period) and were designated as “hot spots.” In addition we calculated a standardized incidence ratio (SIR) with 95% confidence intervals (CI) using the approach by Vandenbroucke [14]. All data was stored, retrieved, and analysed under strict confidential cover using SPSS versus 18. The Ethics Committee of the University of the West Indies approved the study.

## 3. Results

**Figure 1:**
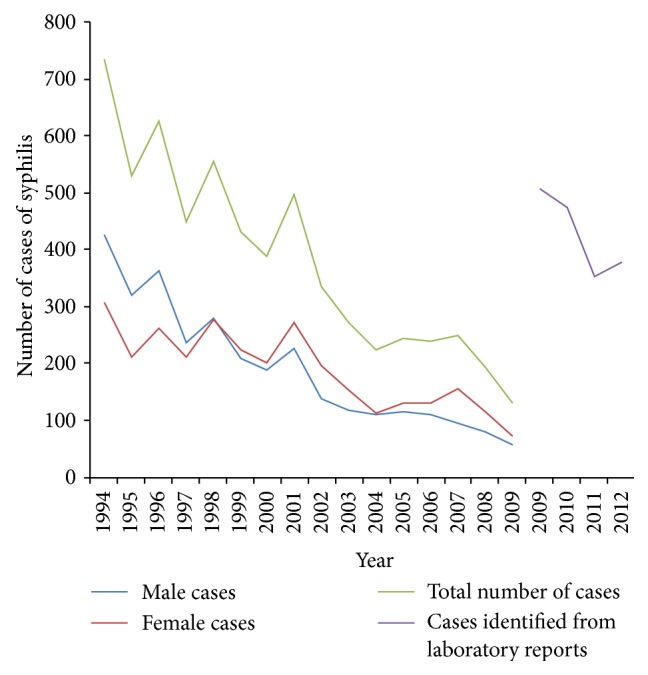
All cases of syphilis reported by the MoH by gender, 1994–2009. All cases identified by review of clinical records at the QPCC, 2009–2012.

Data published by the Ministry of Health (MoH) for the period 2004–2009 revealed a decline in aggregate cases of syphilis during this period (Figure 1). This decline was observed in both males and females. Since there were no published data by the MoH for 2010–2012 we reviewed the laboratory records at the QPCC to determine the number of laboratory confirmed cases for the period (2010–2012). In addition in order to validate the most recent data published by the MoH, that is, 2009, we also reviewed the clinical and laboratory records at the QPCC for 2009. We found the number of cases of syphilis for 2009 was 507 and not 130 as reported by the MoH. Overall the number of cases of syphilis for the period 2009–12 averaged 428, with cumulative incidence rates varying between 39 and 29 per 100 000 population for this period. These rates are similar to those recorded in 1994.

The most recent available data we could obtain is for the period January–April 2013, which may reflect the current situation. In this period there were 64 confirmed cases, 37 (57.8%) males and 27 (42.2%) females. The age group commonly affected was 26–30 years (17, 26.6%). More than half (14, 52%) of the females who tested positive came from samples received from prenatal clinics.

**Figure 2:**
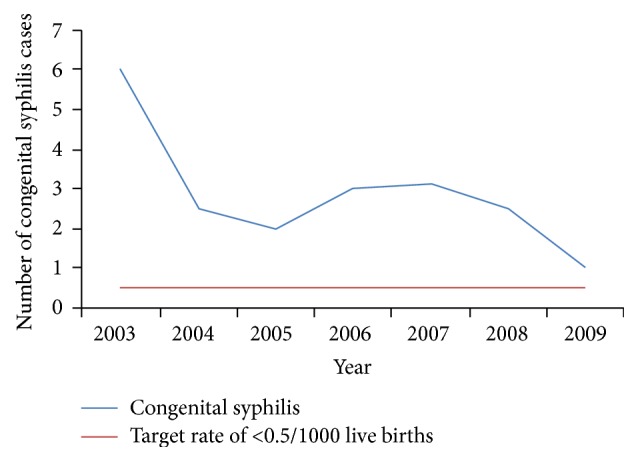
Number of congenital syphilis cases by year, per 1000 live births, 2003–2009.

For the first time data were collected on the sexual orientation of the positive cases. There were more cases of syphilis among heterosexuals (47, 73.4%) than homosexuals (11, 17.2%) or bisexuals (6, 9.4%). These numbers should be interpreted cautiously since it is reasonable to assume (although unknown) that the heterosexual population is much larger than bisexuals or homosexuals. In addition risk taking behaviour, attitudes, and practices among these groups are unknown. Using only data reported by MoH there has been a steady decline in cases of CS (Figure 2). In fact it appears that in 2009 Trinidad had almost reached the target of 0.5 cases per 1000 live births.

**Figure 3:**
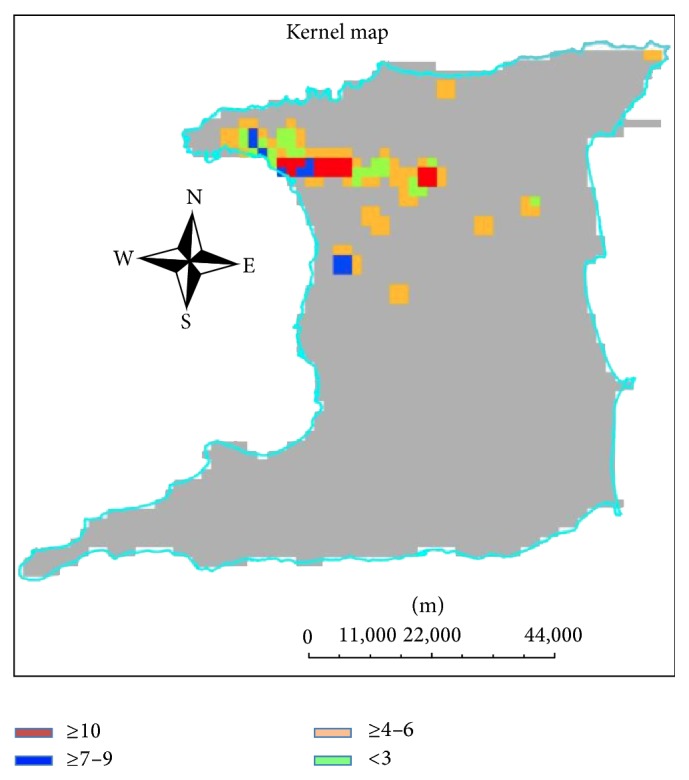
Map of Trinidad with colours depicting density of cases per population density calculated using the Spatial Analyst Kernel Density Tool.

Three hot spots were identified (Beetham Gardens, San Juan/Laventille, and Arima) using the Kernel method (Figure 3). Arima has the second highest population density of 2890 Km^−2^ on the island; both Beetham Gardens and San Juan/Laventille also have high population densities of >1500 Km^−2^. Hence all the areas identified as hot spots were urban and densely populated. We report SIR of 1.62, 95% CI (0.38–2.69).

## 4. Discussion

The major finding of the study was the high levels of occurrence of syphilis still occurring in parts of Trinidad. In fact between 2009 and 2012 the average number of cases per annum was 428 with an incidence rate as high as 39 per 100 000 population. Our data reveal some interesting variations by age and sex. The highest rates of syphilis were predominantly found in urban men and women in their sexually most active years, that is, between the ages of 15 and 35. On average, women become infected at a younger age than men. Consequently, the prevalence of active syphilis is higher among young women than their male peers, a finding that has also been observed for HIV, herpes simplex virus type 2 (HSV-2), and other STDs [15–17]. However in developed countries such as the USA, for instance, there were 46 042 cases of syphilis in 2011 and the rate of primary and secondary syphilis increased by 3.8% among men (from 7.9 to 8.2 cases per 100,000 men) during 2010-2011 [18, 19]. On the other hand, the rate decreased to 9.1% among women (from 1.1 to 1.0 cases per 100,000 women) [20], while in Western Europe rates have fell below 5 per 100 000 in the majority of countries [21–23]. The implication of this finding is clearly that young women are at high risk, and this is of special concern as a high proportion of women become pregnant or commence childbearing before reaching 20 years of age, thus sustaining prenatal transmission and congenital syphilis. These findings support the implementation of educational programs among adolescents to encourage safer sexual behavior and improved treatment seeking behaviour. Based on this evidence we advocate for adolescent sexual health education programs to be implemented in the school curriculum.

Another important finding of the study is the large discrepancy in data reported by the MoH for 2009, that is, 130 reported cases versus 507 cases actually collected from a review of laboratory data. National health data are required for planning and evaluation of service delivery [24–26]. This planning and evaluation is critical in developing countries where the majority of health services are provided through national programs and the limited funds must be used efficiently and effectively [24, 25, 27]. In these settings, high data quality is important to ensure that decisions reflect program needs and direct health professional education priorities [25–28]. Poor data quality not only contributes to poor decisions and loss of confidence in the systems but also threatens the validity of impact evaluation studies [29]. While the study did not focus on the reasons for this discrepancy, several studies have reported inconsistencies in data reporting as well as poor support mechanisms to ensure data quality as it traverses several levels of governmental bureaucracy [30–32].

For example a study done in Nepal found that data obtained from the facility registers were lower than the data reported at the district level, showing a tendency of overreporting to the higher levels [33]. Other studies showed that errors in reporting were due to lack of supervision and feedback from the superior levels as well as inadequate incentives to health workers [30–32]. The quality of data, and consequently of the information system, must be seen in a broader perspective focusing not only on technicalities (data collection tools and the reporting system) but also on support mechanisms.

Another important finding was the widening gap in the occurrence of syphilis among men and women. Historically more men than women contracted the disease but by 1997-98 women began exceeding men and a decade latter this gap was the largest. This finding has two implications; first it is more likely for the disease to be sustained and propagated if women continue to be harbouring the infectious agent and are agents for transmission and secondly the risk of CS remains. While significant gains have been made with congenital syphilis, as of 2009 we are still above the targeted incidence of congenital syphilis, that is, ≤0.5 cases/1,000 live births.

Three hot spots were identified using spatial analysis techniques, as well as a SIR of 1.6. Calculating SIR is recommended as an additional step [34]. The SIR is generally calculated to provide an estimate of the likelihood that an excess of cases exists in the population of concern (hot spots) compared to the general or reference population. The measure is typically interpreted as an excess number of cases in this instance as much as 60%.

There were several limitations to the study mainly the unavailability and difficulty to retrieve all data, which may hinder the exact burden of the disease. In addition because case definitions for congenital syphilis vary widely by country, measuring the attainment of the specific targets of the global initiative may be difficult. In a young child, the possibility of sexual abuse should be considered as a cause of acquired rather than congenital syphilis, depending on the clinical picture; this data could not be determined. Although congenital syphilis includes cases of syphilitic stillbirths we were unaware of the existence of such cases and hence it is not included in the study.

In conclusion the occurrence of syphilis in Trinidad continues to be high; this is against a background in which syphilis screening and treatment can cost less than US $1 per syphilis test and US $0.50 per penicillin dose, and health economists estimate that this is among the most cost-effective public health interventions in existence [35]. In addition elimination of congenital syphilis as a public health problem by 2015 is an important and attainable component of global efforts to achieve the Millennium Development Goals (MDGs) 4 (reduce child mortality), 5 (improve maternal health), and 6 (combat HIV/AIDS, malaria, and other diseases).
